# Value-driven career attitude and job performance: An intermediary role of organizational citizenship behavior

**DOI:** 10.3389/fpsyg.2022.1038832

**Published:** 2022-10-25

**Authors:** Muhammad Babar Iqbal, Jianxun Li, Shuili Yang, Paras Sindhu

**Affiliations:** ^1^School of Economics and Management, Xi’an University of Technology, Xi'an, China; ^2^Department of Business Administration, Sukkur IBA University, Sukkur, Pakistan

**Keywords:** value-driven career attitude, protean career attitude, job performance, organizational citizenship behavior, small and medium enterprises

## Abstract

**Background:**

Value-driven career attitude (VDCA) is considered a dimension of a protean career attitude (PCA). Individuals with this attitude seek out personally meaningful experiences and set their own psychological career success standards. This study investigates the association between value-driven career attitude and job performance. It looks at how organizational citizenship behavior affects the relationship between value-driven career attitudes and job performance.

**Methods:**

A self-reported questionnaire was used to collect data from 400 random employees of SMEs in Pakistan during the early pandemic. We chose Cochran’s formula to determine the appropriate sample size, and PLS-SEM was used to analyze the model. P-O fit and self-determination theory is the theoretical lenses used in this study. The underpinning theories to this study enable the researchers to establish a link between VDCA, OCB, and job performance.

**Results:**

By analyzing a sample of 400 employees from active enterprises, we discover that VDCA contributes to an improvement in job performance. Furthermore, OCB plays an intervening effect in the relationship between VDCA and job performance. Thus, the study provided evidence for the underpinning models of P-O fit and self-determination theory.

**Conclusion:**

This study adds to the body of knowledge by investigating the connections between VDCA, OCB, and job performance in SMEs. The existing literature sheds scant light on these linkages, leaving a gap that this study will address. The current study expands on other themes to provide an in-depth analysis of many under-explored PCA outcomes, which may open up new avenues for future researchers to broaden and strengthen PCA with other constructs.

## Introduction

In the past two decades, organizations have encountered many new difficulties that have profoundly impacted organizational structures and the nature of work. These changes substantially impact career advancement in the workplace (Morley, 2004). Unprecedented occurrences, such as the current COVID-19 outbreak, have forced individuals to reevaluate their employment and professions (Akkermans et al., 2020). Previously, most career studies were based on the conventional career progression paradigm, accentuating full-time, long-term employment with the same organization and a solid commitment to that organization (Valcour and Ladge, 2008). As a career pattern, lifetime employment with a single employer is falling due to the changing career landscape, which is characterized by less loyalty, higher flexibility, and uncertainty in economic and employment connections (Briscoe and Hall, 2006). Employees are now pursuing careers in several organizations, erasing organizational barriers (Kozlowski and Klein, 2000). These developments significantly affect people’s daily lives in the workplace (Harel et al., 2003). Modern career theories explain the career competencies required to manage such contextual transformations effectively. For more than two decades, traditional professional paths gave way to more personalized models, such as the “protean.” Hall (2004) defined the protean concept as a profession in which success standards are psychological and based on personal beliefs, and career management behaviors reflect personal preference rather than the direction of the organization.

The concept of protean career attitude is derived from protean career theory. This concept was inspired by the Greek god Proteus, known for his adaptability and flexibility (Briscoe and Hall, 2006; Waters et al., 2014). The Protean career theory prioritizes individual career management above organizational career management. The theory explains a career type, attitude, or value system that influences a person’s work environment and career choices (Briscoe and Hall, 2006; Waters et al., 2014). A career attitude that is driven by achievement, values, and the desire to defend personal beliefs or principles is classified as “protean” (Segers et al., 2008; Briscoe et al., 2012; Cortellazzo et al., 2020).

Protean career attitude is a positive psychological factor that individuals use to claim their flexibility and advancement in pursuing continuous learning and psychological career accomplishment. This is different from objective career success, which is measured by things like compensation, and position (Hall, 1996, 2004; Arthur et al., 2005). Two sorts of attitudes were recognized by Briscoe and Hall (2006). One of them was a values-driven professional mindset, which posited that a person’s job success is defined and judged based primarily on his or her intrinsic values and beliefs rather than the organization.

People with a protean career attitude are value-driven (Volmer and Spurk, 2011). Individuals exhibit a values-driven attitude when they pursue meaningful work based on their own values of freedom and progress and the goal of psychological success, as opposed to the organization’s values. Their individual values and beliefs influence their career decisions (Hall, 1996; Briscoe and Hall, 2006; Sullivan and Baruch, 2009). Direnzo et al. (2015) and Hall (2004) argue that being “values-driven” does not mean that you always value liberty and freedom of expression. The term may instead be used to emphasize the importance of loyalty, compliance, and devotion (Arnold and Cohen, 2008), along with safety and a way of life (Gerber et al., 2009). Personal values, like one’s identity and self-awareness, are essential to a person. They allow them to choose work that reflects their most important ideas or passions, to be themselves at work, and to find personal fulfillment by adopting subjective motives (Inkson, 2006; Dries, 2011).

A values-driven approach indicates an individual’s heightened awareness of their preferences and serves as the baseline for executing and evaluating decisions (Hall and Mirvis, 1995). Moreover, in the modern workplace, where multiple career paths have replaced the traditional one-way approach to the top, a value-driven mentality is a prerequisite for an individual to recognize the distinctiveness of his or her career life (Richardson, 2000). This career path is marked by job mobility in multiple directions and the use of an ample range of transferable professional credentials in flexible arrangements with different organizations (Baruch, 2006; Clarke, 2009).

According to several studies, being career-proactive and self-driven increases job happiness and perceived professional prosperity (Cooper-Hakim and Viswesvaran, 2005; Cabrera, 2009). Values-driven individuals were more likely to be proactive, engrossed in learning, and open to new experiences, but their association with social capital was not statistically significant (Li et al., 2022). Katz and Kahn (1966) argues that developing and defining identity depends on the expression of values. New evidence suggests that a value-driven career outlook strongly indicates internal and external confidence in one’s employability (Lin, 2015). As a result, career researchers have changed their focus from organizational to personal career management, with a few notable exceptions (Baruch and Peiperl, 2000; Quigley and Tymon, 2006; Abele and Wiese, 2008). Research on unemployment also shows a connection between the PC’s values-driven component and self-esteem since maintaining one’s sense of self while unemployed is linked to psychological well-being (Cassidy, 2001).

Segers et al. (2008) discovered an absolute correlation between age and gender and a values-driven attitude, with men having higher values-driven as opposed to women and values-driven career management findings rising with age. In contrast, Mainiero and Sullivan (2005) find that women are more values-driven, and differences may exist for people who are further along in their careers. In this regard, Inceoglu et al. (2008) claimed that females are more motivated by job security and are choosing jobs that allow them to be successful on their own terms rather than by objective metrics of career accomplishment like money, status, and promotion (Heslin, 2005). Values-driven and mobile preferences should be advantageous in a career climate that is more employee-centric (Li et al., 2022). Few studies also showed that affective commitment is inversely correlated with a value-driven career orientation (Alonderienė and Šimkevičiūtė, 2018). Çakmak-Otluoğlu (2012) highlights the conflict between normative commitment and value-driven career management. He also claimed that hesitant individuals about their personal values are more normatively devoted to their organizations. In contrast, few studies found that the values-driven PCO negatively impacted psychological well-being (Rahim and Zainal, 2015; Li, 2018).

Person-organization (P-O) fit theory and self-determination theory can be stated along these lines to conceptualize and establish the relationship between VDCA, OCB, and job performance. The compatibility of values and expectations between individuals and organizations is called P-O fit. P-O fit is a building element of the P-E fit construct (Caplan, 1987). Values are an essential characteristic that can be directly and meaningfully compared between individuals and organizations (Cable and Judge, 1997). According to Argyris (1957), an individual’s organizational behavior results from an interaction between the person and the organization (Verquer et al., 2003). Most research on P-O fit has focused on the congruence between organizational value patterns and human value behaviors (Kristof, 1996). Value congruence is now generally recognized as the operationalization of P-O fit (Kristof-Brown et al., 2005). When highly valued personnel share the same values as the organization, they will adhere to the firm’s values (Gubler et al., 2014; Li et al., 2022). This value congruence is expected to positively impact individual performance (Kristof-Brown et al., 2005; Butt et al., 2020). P-O fit is essential for organizations because there are strong links between it and organizational citizenship behaviors (Tziner, 1987), work attitudes (Dawis and Lofquist, 1984; Cable and Parsons, 2001), and job performance (Cable and Parsons, 2001).

For understanding VDCA and job performance, a comprehensive framework is provided by self-determination theory. Self-determination theory claims that intrinsic values and motivations can lead to fulfillment since they reflect psychological development and self-actualization, which sets them apart from extrinsic motivations (Deci and Ryan, 1985). VDCA is also associated with inner feelings of contentment and self-actualization (Hall and Moss, 1998). Kuvaas (2009) found that the intrinsic motivation of public sector employees was substantially connected with their self-reported job performance and knowledge sharing.

Although the protean career has been studied extensively, there is little empirical evidence to support it because it is still in the preliminary phases of establishing generic roots (Hall, 2004; Granrose and Baccili, 2006; De Vos and Soens, 2008). Prior studies have paid little consideration to the relationship between VDCA, OCB, and job performance. This study intends to establish empirical evidence on these links by utilizing this gap, particularly in developing nations such as Pakistan. Despite significant research on SMEs in both established and emerging economies (Eze and Okpala, 2015; Manzoor et al., 2021), no study has been focused on the relationship between VDCA, OCB, and job performance in the SME sector. Furthermore, due to the individualistic nature of Western culture, the concept of PCA (VDCA and SDCA) has gained the most attention (Tschopp et al., 2014). As a result, there was a call for empirical PCA research in a non-American culture to investigate potential divergence in career attitudes due to communal and civilizing differences (Sullivan and Baruch, 2009). Consequently, this study explored the impact of a value-driven career attitude on job performance, with OCB serving as a mediator in the SME sector. This study focuses on current VDCA knowledge as people’s interest in protean careers grows (Park et al., 2022), particularly during the pandemic.

The success of SMEs is frequently used as a barometer of economic growth (Veskaisri et al., 2007). Small businesses’ efficiency and competitiveness are crucial to economic growth (Beaver, 2003). These economies prioritize the SME sector regarding economic growth and job generation (Dalrymple, 2004; Laudal, 2011; Mehmood et al., 2019). In emerging nations like Pakistan, SMEs account for 80% of employment, 40% of GDP, and around 30% of exports (SMEDA, 2005, 2007, 2008-2009). SMEs still have difficulty keeping skilled people, so finding, training, and retaining talent is becoming increasingly important. Organizations must assist employees in enhancing their performance by providing the necessary developmental resources and support. In this setting, SMEs should develop career advancement strategies that are adapted to the needs of the workforce and effective at retaining outstanding employees (Star, 2020).

This study investigates the association between a value-driven career attitude and job performance. It looks into how organizational citizenship behavior mediates between value-driven career attitudes and job performance. It reviews the literature on VDCA, OCB, and job performance, as well as the significance of such phenomena in SMEs in developing nations where the research was conducted. The four hypotheses provided in this research are examined using structural equation modeling. The following stage evaluates the pertinent literature, describing the research methods and findings. On the basis of the results and limitations of this study, the implications and the potential for additional research are underlined.

## Literature review

This section investigates the existing literature on value-driven career attitudes, OCB, and job performance from several theoretical and relational vantage points.

### VDCA and job performance

According to Motowidlo (2003), a person’s job performance is the total expected value to the organization of the discrete behavioral episodes a person does over a certain amount of time. The concept of performance has developed over time (Campbell et al., 1993; Welbourne et al., 1998; Johnson, 2003). For many years, researchers have judged the performance of individuals based on how well they carry out the responsibilities outlined in their job descriptions (Locke and Latham, 1990; Ilgen and Pulakos, 1999). Recently, however, job performance has been evaluated based on specific behaviors linked to the features and surroundings of the workplace (Pulakos et al., 2000; Griffin et al., 2007). This transformation occurred as the organizational environment shifted from stable to dynamic (Griffin et al., 2007). As a result, rather than being evaluated on their ability to do job, employees are assessed on how they behave in their roles (Ilgen and Pulakos, 1999).

Over time, the nature of work has evolved from being relatively static to becoming fluid. Several studies have examined how different organizational traits affect performance to determine how supervisors may enhance the performance of their employees. Hall (2004) indicates that an individual with a strong PCA (VDCA and SDCA) may be a higher performer due to his or her intrinsic motivation and ambition for psychological achievement (Hall et al., 2018), which can result in a tremendous performance. Baruch (2014) determined the strong relationship between PCA (VDCA and SDCA) and task performance. According to De Vos and Soens (2008), PCA is a potent predictor of professional growth. Briscoe et al. (2012) and Baruch (2014) discovered a correlation between self-reported individual performance metrics and protean career inclinations. Individuals can improve their job performance and self-actualization to achieve their objectives, influencing professional decision-making and exploration (Hall, 2004; Waters et al., 2015).

Constructive work attitudes and innovation positively impact job performance in organizations with employee-centered designs (Christensen et al., 2009). Research has also shown that spending on human capital improves job performance, employability, sustainability in the workforce, and the capacity to adjust to fluctuating market working conditions (Casuneanu, 2011; Manzoor et al., 2019). There is a substantial correlation between P-O fit and job performance (Cable and Parsons, 2001), which makes it crucial for organizations. Employees with VDCA are proactive, and proactivity was found to be a component of job function performance (Griffin et al., 2007). They obtain information on potential work possibilities, career interests, talents, and abilities by soliciting comments on their job performance (Weng and McElroy, 2010; Li, 2018). A positive and confident outlook on one’s protean career (VDCA and SDCA) has been shown to have a statistically significant, positive interaction with work excitement and proactivity (Seibert et al., 1999; Baruch, 2014). Career proactivity is empirically linked to job success, career happiness (Butt et al., 2020), career advancement, and job performance (Locke et al., 1984; Seibert et al., 2001; Arthur et al., 2005; Volmer and Spurk, 2011).

We feel that in today’s highly competitive business environment, a person with a robust value-driven attitude will be more likely to achieve outstanding job performance, as these attitudes are closely associated with job satisfaction and productivity. This ubiquitous logic postulates as

*H1:* Value-driven career attitude has an impact on job performance.

### VDCA and OCB

OCBs are employee actions that contribute to and improve the psychological and social work environment. These actions aren’t required by a structured pay system and are meant to help the organization do its job well (Luthans, 2005). Williams and Anderson (1991) divided OCB into two types and classified them as OCBI and OCBO. OCBI refers to behaviors that directly benefit specific individuals within an organization and, as a result, indirectly contribute to organizational effectiveness (Williams and Anderson, 1991; Organ, 1997; Organ and Paine, 1999; Lee and Allen, 2002; Dalal, 2005; Niroula and Chamlagai, 2020). OCBO is a term for actions that a person takes that could improve the performance of an organization as a whole (Williams and Anderson, 1991; Organ, 1997; Organ and Paine, 1999; Dalal, 2005). The progress of an organization is dependent not just on employees’ performance inside their assigned roles but also on their performance outside of those roles (Podsakoff et al., 1997). Benefits from OCBs may accrue to the organization as a whole or an individual employee (Williams and Anderson, 1991). Regardless of the OCBs’ intended audience, organizations that can motivate their employees to make this extra effort will have a more robust workforce and a significant competitive advantage (Bolino and Turnley, 2003). Several researchers have emphasized the importance of OCB for human resources because it correlates with the efficient operation of organizations (Hui et al., 2004; Shih and Chen, 2011) and partially incorporates in-role performance in assessment appraisal (Presti et al., 2019).

OCB has garnered the majority of research focus (Smith et al., 1983). Previous empirical studies have examined the affiliation between OCB and a range of other variables, including employee satisfaction, job commitment, turnover intentions (Huak et al., 2015), intention to stay (Priyanka, 2016), quality management, and organizational performance (Aslefallah and Badizadeh, 2014). Previous studies exploring the relationship between these concepts and other factors found that PCA (VDCA and SDCA) was positively associated with OCB. (Jawahar et al., 2008; Presti et al., 2019). A study conducted in Portugal relating autonomous career orientations with OCBs revealed that individuals with a high PCA exhibit more OCBs (Rodrigues et al., 2015). The association analysis results fallout a strong link between the traits of protean jobs and the OCB (Joshi et al., 2021).

The empirical research on the link between VDCA and OCB is in its infancy (Rowe, 2013; Alok and Rajthilak, 2021; Joshi et al., 2021). Thus, it is hypothesized that a value-driven career attitude would have a favorable influence on OCB.

*H2:* Value-driven career attitude would positively influence OCB.

### Value-driven career attitude, OCB, and job performance

Existing research demonstrates that PCA (VDCA and SDCA) professionals benefit themselves and their organizations by accomplishing greater levels of intrinsic and extrinsic career triumph through exemplary job performance. A protean career is linked to better job performance, higher OCBs, and higher organizational involvement (Rodrigues et al., 2015), all of which lead to better organizational performance (Hall, 2004). Employee advancement is linked with enhanced performance, OCB, organizational commitment, job satisfaction, and reduced intention to leave the organization (Birdi et al., 1997; Ellinger, 2004; Benson, 2006).

Organizational citizenship behaviors (OCBs) have long been marked as an important component of individual success and performance (Dunlop and Lee, 2004). Several studies investigate the significance of OCB in determining the association between human resource management approaches and performance (Sun et al., 2007). Researchers have discovered a strong link between P-O fit, organizational citizenship behavior, and job performance in the past (Tziner, 1987; Cable and DeRue, 2002; Iplik et al., 2011). Chien (2003) argues that OCB’s incorporation into the workplace can boost productivity on both an individual and companywide scale. Previous research has assessed the impacts of OCB on performance, customer service and satisfaction, sales revenue, and capital sufficiency (Organ et al., 2011). Individual OCB contributes to enhanced organizational performance and operational effectiveness (Choi, 2009; Organ et al., 2011). Individuals can improve their job performance and self-actualization to achieve their objectives, subsequently influencing professional decision-making and exploration (Hall, 2004; Waters et al., 2015). OCB has been found to be a conclusive predictor of job performance in previous research (Podsakoff et al., 2000; Harwiki, 2016; Bagyo, 2018; Hidayah and Harnoto, 2018; Lestari, 2018; Soumyaja and Kamalanabhan, 2022) and a significant contributor to both organizational effectiveness and social relationships inside the firm (Organ et al., 2005). Previous research discovered that OCB influenced high-and low-performance respondents (Callea et al., 2016; Laski and Moosavi, 2016; Basu et al., 2017; Huang et al., 2017). OCB across individuals enhances organizational effectiveness (Choi, 2009).

In addition, no prior research has evaluated the association between OCB in VDCA and job performance mediation. Based on the above facts, we have sought to assess and explain the relationship between value-driven career attitude and job performance, considering OCB as a mediating variable. Thus, it is hypothesized that:

*H3:* There is a positive relationship between OCB and job performance.

*H4:* OCB plays a mediating role between VDCA and job performance.

**Figure 1:**
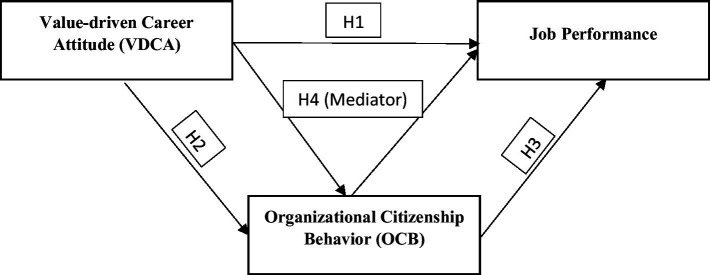
Conceptual model.

Figure 1 depicts the conceptual model described in the study's theoretical framework.

## Methodology

### Sampling

The sample for this study is comprised of Pakistani professionals working for SMEs. In order to limit sampling error, it is necessary to designate an adequate sample size in survey research (Hair et al., 2010a; Siyal et al., 2019). To this end, we chose Cochran’s formula (Cohen, 1992; Siyal et al., 2019). The Cochran’s formula is appropriate for use in situations where the population size is unknown but the number is high (Chaokromthong and Sintao, 2021). A minimum sample size of 385 was acquired based on criteria at a confidence level of 95% with a precision of 5% (plus or minus; *z* value was 1.96). In order to address the issue of inappropriate responses, a minimum of 500 samples must be collected. A large sample size ensures the accuracy of structural equation modeling (Siyal et al., 2019). Due to its diversity and the Cochran formula, stratified sampling is a valid method for determining the sample’s representation of the larger population (Stringer et al., 2011). Based on a number of tests, it was decided that the research’s sample was a good representation of the whole population, with a reliability of 0.90 and a good level of consistency across all questions:


$$\begin{array}{c} n_{0}=\frac{Z^{2}\;pq}{e^{2}}\\ =\left[{\left(1.96\right)}^{2}\left(0.5\right)\;\left(0.5\right)\right]/{\left(0.05\right)}^{2}\\ =385 \end{array}$$

### Data collection procedure

For this study, 500 employees from active SMEs in Pakistan were chosen from all administrative departments. Out of the 500 eligible respondents, 450 questionnaires were returned. Due to insufficient information provided by respondents, 50 questionnaires were denied, whereas 400 completed questionnaires were utilized for data analysis in this study. This study admitted 271 males (67.75%) and 129 females (32.25%). The ages of key informants ranged from under 25 (63), 26 to 35 (183), 36 to 45 (107), and over 45 (47), with percentages of 15.75, 45.75, 26.75, and 11.75, respectively. In this study, career levels ranging from supervisor/worker (24, 6%), middle-level manager (269, 67.25%), manager (105, 26.25%), and owner/CEO (2, 0.50%) were counted. Similarly, respondents with basic/secondary level (20), undergraduate (156), Master’s (221), and Ph.D. (3) degrees had 5%, 39%, 55.25%, and 0.75%, respectively. Tables 1, 2 depict the demographic diversity and characteristics of the study population in detail.

**Table 1:** Respondents’ characteristics.

Respondents characteristics	Frequency	Percent
*Gender*		
Men	271	67.75
Women	129	32.25
Total	400	100
*Age group*		
Under 25	63	15.75
26–35	183	45.75
36–45	107	26.75
Over 45	47	11.75
Total	400	100
*Education*		
Secondary or basic	20	5
Undergraduate	156	39
Masters	221	55.25
PhD	3	0.75
Total	400	100
*Position*		
Supervisor/Worker	24	6
Middle manager	269	67.25
Manager	105	26.25
Owner/CEO	2	0.50
Total	400	100

**Table 2:** Enterprise characteristics.

Enterprises characteristics	Frequency	Percent
*Type of enterprise*		
Educational sector	170	42.50
Health and care services	90	22.50
Other services	140	35
Total	400	100
*Size of enterprise*		
5–30	210	52.50
31–60	134	33.50
61–99	32	8
100 or higher	24	6
Total	400	100
*Age of enterprise (in years)*		
1–5	70	17.50
6–10	168	42
11–15	112	28
15 or higher	50	12.50
Total	400	100

### Measures

Standardized measurements were used to collect information on value-driven career attitudes, organizational citizenship behaviors, and job performance. Responses were recorded on a five-point Likert scale.

#### Value-driven career attitude

The VDCA was tested using the six items of the PCA scale created by Briscoe et al. (2006). On a five-point Likert scale, individuals indicate the extent to which they believe they are in charge of their values (e.g., “I will follow my own advice if my employer asks me to do something against my values”). The scale ranges from 1 (to little or no) to 5 (to a great extent). This values-driven scale measures the degree to which individuals follow their careers instead of organizational values (Volmer and Spurk, 2011).

#### Organizational citizenship behavior

The OCB scale developed by Lee and Allen (2002) was utilized to collect data for this research. This measurement consists of 16 elements evaluating the OCBI and OCBO. However, items 3, 14, and 16, namely OCBI3, OCBO6, and OCBO8, were dropped due to low factor loadings. As a rule of thumb, 20 percent of the total items can be eliminated (Hair et al., 2010b). Questions were graded on a five-point Likert scale, with 1 indicating never and 5 indicating always.

#### Job performance

The scale developed by Koopmans et al. (2013) was adopted to evaluate job performance. The scale has multiple items. Seven items that gage task performance and contextual performance were gathered (Motowidlo, 2012). According to Borman and Motowidlo (1993), two key components determine job performance (task performance and contextual performance). Questions were asked using a Likert, where 1 meant never, and 5 meant always.

## Results and discussion

In this study, the theoretical model was examined using PLS-SEM. PLS-SEM is preferred above other standard multivariate techniques (Haenlein and Kaplan, 2004; Ren and Hussain, 2022). PLS-SEM offers a statistically precise evaluation based on a bootstrapping method that yields standard errors for route coefficients (Preacher and Hayes, 2008; Martínez-Martínez et al., 2017). Several assumptions were examined, including multicollinearity, normality, and common method variance (Tabachnick et al., 2007; Hair et al., 2010a; Umrani et al., 2018; Zafar et al., 2021). The researchers then examined the data’s reliability, validity, and structural path. After analyzing the measurement model, the structural model was evaluated using Partial Least-Squares Structural Equation Modeling (Henseler et al., 2009; Hair et al., 2010a; Rahman and Karim, 2022). The model, which included VDCA, OCB, and job performance, was evaluated in two steps: a measurement model and a structural model (Hair et al., 2014).

### Measurement model assessment

In order to evaluate the measurement model, it is crucial to quantify each concept’s reliability, internal consistency, convergent validity, and discriminant validity (Hair et al., 2010a). We employed PLS-SEM for this aim because scholars in a range of domains widely accept it. Due to its new criteria for critical data analysis, it is ideally suited for this research (Hair et al., 2019).

#### Individual item reliability

The factor loadings of each item in a construct are required to assess the reliability of individual items (Hulland, 1999; Duarte and Raposo, 2010). Hair et al. (2019) proposed that maintaining an item with a value equal to or above 0.5 is considerable. In this investigation, all outer loadings were more than 0.7 (see Table 3), indicating that the individual item reliability criteria were fulfilled.

**Table 3:** Factor loadings and variance inflated factor.

Construct	Item	Loading	VIF
Value-driven career attitude	VDCA1	0.706	1.676
	VDCA2	0.792	2.008
	VDCA3	0.825	2.139
	VDCA4	0.822	2.239
	VDCA5	0.775	1.829
	VDCA6	0.768	1.814
Organizational citizenship behavior	OCBI1	0.790	2.356
	OCBI2	0.734	1.903
	OCBI4	0.755	2.105
	OCBI5	0.743	2.046
	OCBI6	0.772	2.343
	OCBI7	0.719	1.965
	OCBI8	0.744	2.149
	OCBO1	0.792	2.463
	OCBO2	0.792	2.415
	OCBO3	0.754	2.122
	OCBO4	0.781	2.423
	OCBO5	0.758	2.138
	OCBO7	0.788	2.279
Job performance	CP1	0.764	1.885
	CP2	0.785	2.070
	CP3	0.795	2.154
	TP1	0.780	2.024
	TP2	0.834	2.544
	TP3	0.744	1.919
	TP4	0.813	2.160

VIF, variance inflated factor.

#### Internal consistency

The average variance extracted (AVE) was utilized to determine convergent validity (Fornell and Larcker, 1981; Bagozzi et al., 1991; Siyal et al., 2019), and all AVE values for latent variables were >0.50. Similarly, researchers evaluated internal consistency reliability by examining composite reliability (CR) scores with a minimum verge of 0.70 and Cronbach’s alpha with a minimum verge of 0.70 (Hair et al., 2016; Siyal et al., 2019; Umrani et al., 2020). The composite reliability coefficients for each construct in this study are displayed in Table 4. This indicates that construct internal consistency reliability is adequate (Bagozzi et al., 1991). The variance inflated factor (VIF) was utilized to evaluate the research design and collinearity bias. Ringle et al. (2015) proposed a VIF verge of 5 or less as the reciprocal of tolerance (Table 3).

**Table 4:** Mean, SD, CA, CR, and AVE.

Constructs	Mean	SD	CA	CR	AVE
Value-driven career attitude	3.77	0.82	0.873	0.904	0.612
Organizational citizenship behavior	3.66	0.83	0.940	0.948	0.583
Job performance	3.86	0.79	0.898	0.920	0.621

SD, standard deviation; CA, Cronbach alpha; CR, composite reliability; AVE, average variance extracted.

#### Convergent validity

The average variance extracted (AVE) was offered to assess convergent validity (Fornell and Larcker, 1981). A value of 0.5 or above may be used to demonstrate the convergent validity of a specific variable by adhering to Chin (1998) criterion. According to the AVE values in Table 4, this study obtained an AVE value above the 0.5 thresholds, indicating good convergent validity.

#### Discriminant validity

Fornell and Larcker (1981) state that discriminant validity can be measured using an AVE value of 0.5 or higher. To demonstrate discriminant validity, the square root of the AVE should also be greater than the correlations between the latent components. As indicated in Table 4, all latent variable AVE values were higher than the cutoff. The correlations between the latent components were smaller than the square root of AVE, as seen in Table 5. Consequently, all metrics indicate that the current investigation’s discriminant validity is sufficient.

**Table 5:** Discriminant validity.

Constructs	1	2	3
JP	0.788		
OCB	0.601	0.764	
VDCA	0.696	0.486	0.782

### Structural model assessment

When assessing a model’s potential usefulness, the predictive capability is measured by R2 (Sarstedt et al., 2014). The R2 value indicates the amount of variance in the dependent variable(s) that can be explained by the predictor variable(s) (Hair et al., 2006, 2014; Umrani et al., 2018). According to Umrani et al. (2018), the conditions under which a particular study is conducted determine the acceptable amount of R2 value. According to Chin (1998), an R2 value of 0.60 is considered strong, 0.33 is considered modest, and 0.19 is viewed as weak. However, according to Falk and Miller (1992), an R2 of 0.10 is considered adequate. Based on our data, the coefficient of determination for OCB is = 0.237, and the coefficient for job performance is = 0.574 (Table 6). The value is sufficiently higher than the minimal permissible cutoff, according to Falk and Miller (1992). The square root of the residuals provides an absolute measure of fit, with a value of zero indicating a perfect fit. The SRMR measures the mean squared discordance between observed and implicit correlations in the model. The results show that the SRMR = 0.044 and the NFI = 0.907 are significant; nonetheless, the NFI fit was still satisfactory since it was >0.8 (Zikmund, 2003). Table 6 shows that the study supports the assumption that an SRMR value of 0.08 or less is acceptable (Hu and Bentler, 1998).

**Table 6:** Model fit summary.

SRMR	0.044	R2 for OCB = 0.237
d_ULS	0.682	
d_G	0.266	R2 for JP = 0.574
Chi-square	591.076	
NFI	0.907	

SRMR, standardized root mean squared residual; d_ULS, the squared Euclidean distance; d_G, geodesic distance; NFI, normed fit index.

**Figure 2:**
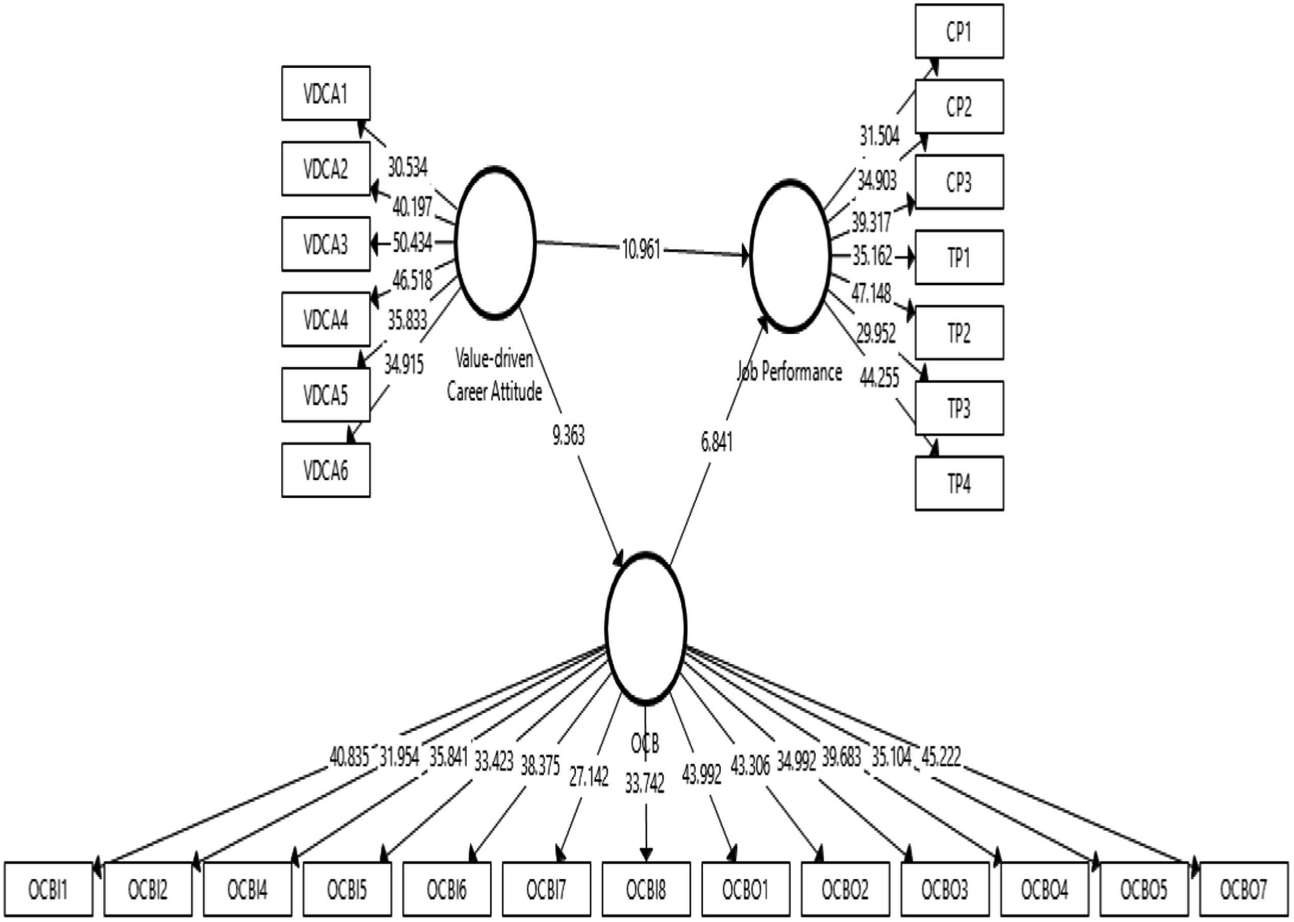
Hypothesis results.

This study used the bootstrapping technique with 5,000 bootstrap samples and a sample size of 400 to determine the significance of the path coefficients (Hair et al., 2010a, 2011, 2014). Table 7 and Figure 2 show that full estimates were obtained using this structural model with statistics. First, H1 predicted that VDCA is strongly associated with job performance. The results of Table 7 and Figure 2 confirmed a confident relationship between VDCA and job performance at a 1% significance level, (*β* = 0.529, *t* = 10.855, *p* = 0.000). As a result, H1 has been confirmed.

**Table 7:** Structural model.

Hypothesis	Relationship	Beta	SE	*t*-Value	Value of *p*	Decision
H1	VDCA → JP	0.529	0.049	10.855	0.000	Supported
H2	VDCA → OCB	0.486	0.053	9.230	0.000	Supported
H3	OCB → JP	0.343	0.051	6.785	0.000	Supported
H4	VDCA → OCB → JP	0.167	0.034	4.873	0.000	Supported/partial mediation

Second, we postulate that VDCA has a positive effect on OCB. The outcomes demonstrate a statistically significant association between VDCA and OCB (*β* = 0.486, *t* = 9.230, *p* = 0.000). A second hypothesis, H2, was also tolerated. In hypothesis 3, OCB has a positive impact on job performance. Positive correlations between OCB and job performance were found, as evidenced by the relationship’s *β* = 0.343, *t* = 6.785, and *p* = 0.000 coefficients. The results showed a significant VDCA-OCB-job performance association (*β* = 0.167, *t* = 4.873, *p* = 0.000), proving the fourth hypothesis.

If an indirect path traverses an intermediary construct (mediator), then, according to Baron and Kenny (1986), the mediator serves as a link between independent and dependent constructs. The model is statistically significant if both paths from the independent variable to the dependent variable are significant. If both the direct and indirect paths from the independent variable to the dependent variable are substantial, this indicates some degree of mediation (DeJong et al., 2019). Complete mediation occurs when the direct path is negligible, and the indirect path is significant. The research demonstrates that OCB has a partial mediating impact, supporting Hypothesis 4. Concur that complementing mediation occurs when both direct and indirect pathways are essential and considerable. The results of this study are shown in Table 7.

### Discussion

The primary goal of this study was to investigate the effect of a VDCA on job performance. The data indicates a substantial correlation between VDCA and job performance. These findings confirm previous research revealing that PCA professionals benefit themselves and their organizations by accomplishing tremendous intrinsic and extrinsic career success through exemplary job performance (Sultana and Malik, 2019). Hasan and Altaee (2020) found that bringing people from different career paths together can improve the alignment of high performance in the workplace.

The VDCA appears to promote OCB, resulting in a substantial return for the organization and correlating with an abundance of empirical evidence in the field of previously analyzed studies. According to the study, proactive VDCA employees are likelier to engage in workplace development drives (Jawahar and Liu, 2016). Sturges et al. (2002) found that workers who take charge of their careers are better organizational citizens.

OCB is increasingly recognized as a crucial organizational asset and positively impacts organizational atmosphere and operations (Pletzer et al., 2021). Regarding the hypotheses, this study produces positive results corresponding to evidence already documented in the literature. Individuals with a high VDCA were found to engage in more organizationally healthy behaviors. In contrast, an increase in VDCA was linked with a greater level of commitment and a positive indirect link to extra-role behaviors. Employees with OCB levels above a certain threshold have a positive attitude toward their work and the organization, which translates into more productive work output.

## Conclusion

In conclusion, contemporary careers and career selection in the global economy are chaotic and fraught with uncertainty for both individuals and organizations. The potential links between modern professional mindsets and job success have received little consideration from researchers. Evaluating the impact of contemporary careers in developing nations where job security is significant stands out as a challenge. Nonetheless, this study is the first step in defining PCA dimensions as independent constructs and legitimizing them in human and organizational contexts by examining developing nations. An organization should immediately focus on developing career concepts and how they affect the organization and the outside world.

The current study confirms that VDCA significantly contributes to improved job performance and that OCB mediates the relationship between VDCA and job performance. Consequently, firms that invest in performance enhancement to meet individual and organizational objectives should prioritize developing career concepts. This research aids in comprehending the mechanism by which the emerging attitude affects human capital, which is vital for effective career management and the formulation of suitable HR policies (Baruch, 2014). It’s essential to measure how employees feel about their occupations in response to various work designs and organizational support initiatives. This study offers new insights into how enhancing organizational development across the board in developing nations can be accomplished by managing employees effectively in new career management. The two-way communication made possible by PCA allows employers and workers to collaborate on projects and share ideas for improving the organization and the workplace. Which, in the end, aids businesses in reaching their intended markets and forming strategic partnerships.

### Limitations and future work

Our findings provide a solid foundation for future research on new career concepts. Similar to the novel contribution to the career literature, this study includes limitations that should be addressed in future studies. One of the primary drawbacks is that it is limited to the occupational field, namely SMEs, restricting the findings’ external validity and generalizability. In the future, the model of this study could be examined with samples from other areas, such as new or emerging markets or more well-known companies, to make our results more applicable to a broader range of situations. Future research can further explore this new career orientation concept by examining it with other variables. In addition, the study encourages replication in various cultural contexts and with diverse samples to increase generalizability. The COVID-19 pandemic is also a limitation because it occurred while collecting data. This means that the findings and effects of future studies may differ from those of this research.

### Theoretical and practical implications

Due to the individualistic aspect of Western culture, the concept of PCA has garnered crucial attention (Tschopp et al., 2014). Consequently, there was a demand for empirical PCA research in non-American contexts to study potential disparities in career attitudes related to communal and civilizing characteristics (Sullivan and Baruch, 2009). The researchers empirically tested the hypothesis and discovered that VDCA significantly affects job performance and that OCB acts as a mediator between VDCA and job performance. The significance of the study was achieved, as stipulated in the conceptual model; hypotheses are in accordance with research objectives, and research objectives are in line with research questions.

In a world where change occurs rapidly, the study could assist HRD experts and managers in helping their employees to plan their careers and align their job performance goals with their professional goals and triumphs. The resultant creation of HR policies that promote evolving career conceptions will aid in preventing employee turnover. On the other hand, organizational career management rules that permit self-management will assist protean talented workers in maintaining their jobs and achieving their goals, thereby preventing them from leaving their current positions. These new findings shed light on how career flexibility impacts various organizational divisions. Youth unemployment is a critical challenge facing Pakistani society. However, organizations now have a more significant duty to investigate the human attitudes and behaviors influencing how employees perform and view their professional development.

## Data availability statement

The original contributions presented in the study are included in the article/supplementary material, further inquiries can be directed to the corresponding author.

## Ethics statement

This research has been approved by the Research Ethics Committee of Xi’an University of Technology. Written informed consent to participate in this study was provided by the participants.

## Author contributions

All authors listed have made a substantial, direct, and intellectual contribution to the work and approved it for publication.

## Conflict of interest

The authors declare that the research was conducted in the absence of any commercial or financial relationships that could be construed as a potential conflict of interest.

## Publisher’s note

All claims expressed in this article are solely those of the authors and do not necessarily represent those of their affiliated organizations, or those of the publisher, the editors and the reviewers. Any product that may be evaluated in this article, or claim that may be made by its manufacturer, is not guaranteed or endorsed by the publisher.
